# Supplementary figures and images for: A-Kinase Anchoring Proteins Diminish TGF-β1/Cigarette Smoke-Induced Epithelial-To-Mesenchymal Transition
Source: Cells. 2020 Feb 3;9(2):356. doi: 10.3390/cells9020356 (PMC7072527; doi:10.3390/cells9020356)

# Supplementary figure1

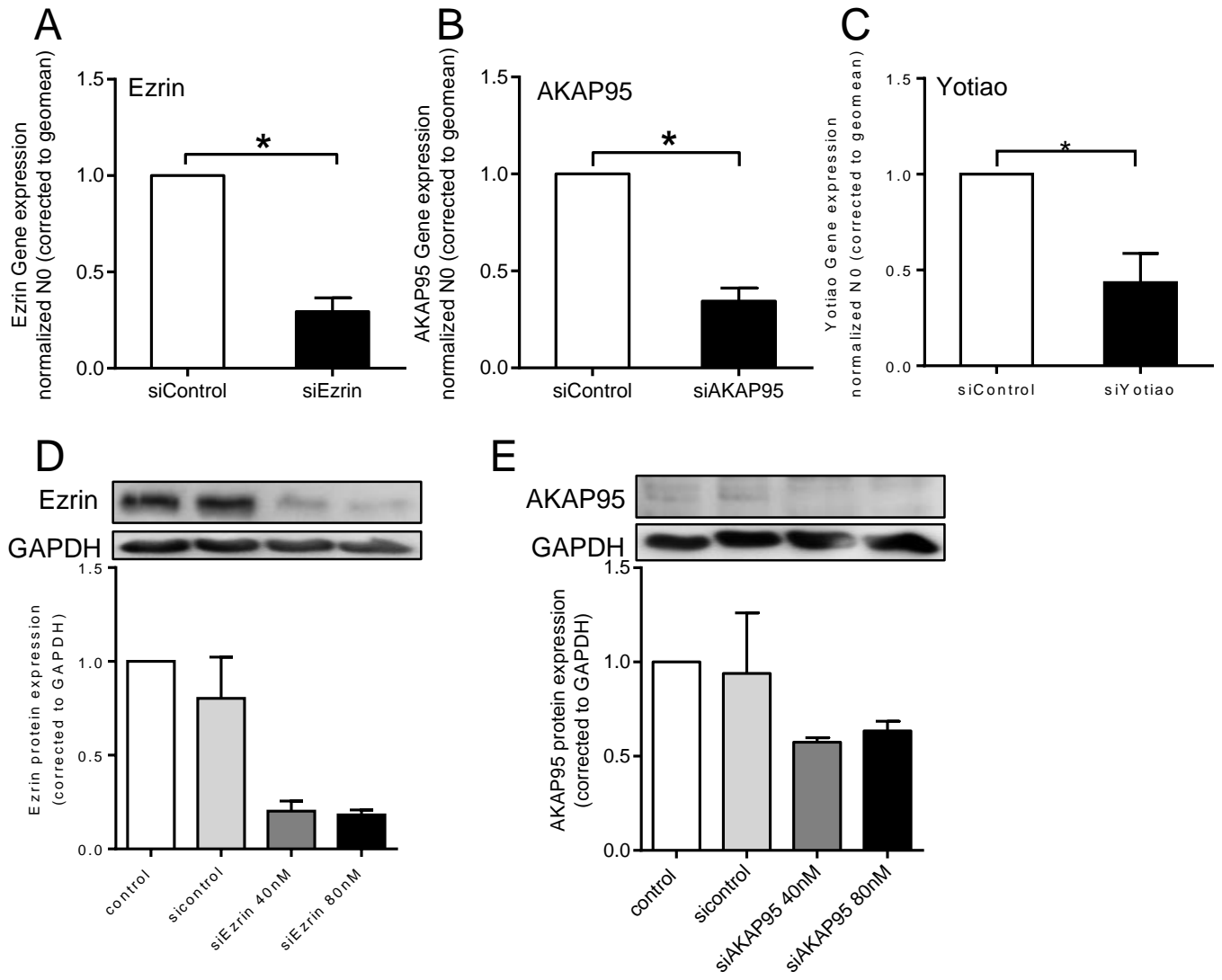

A

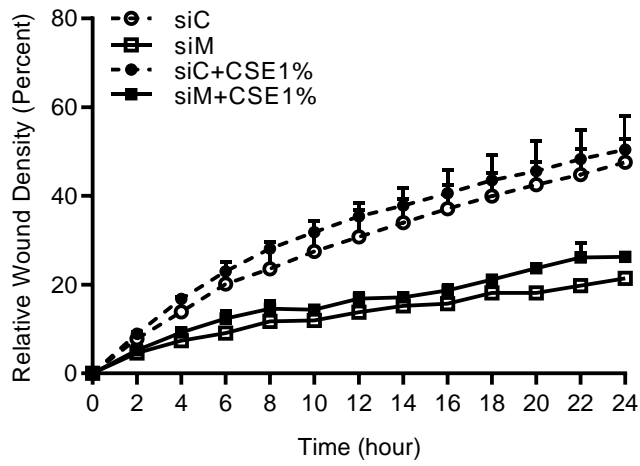

B

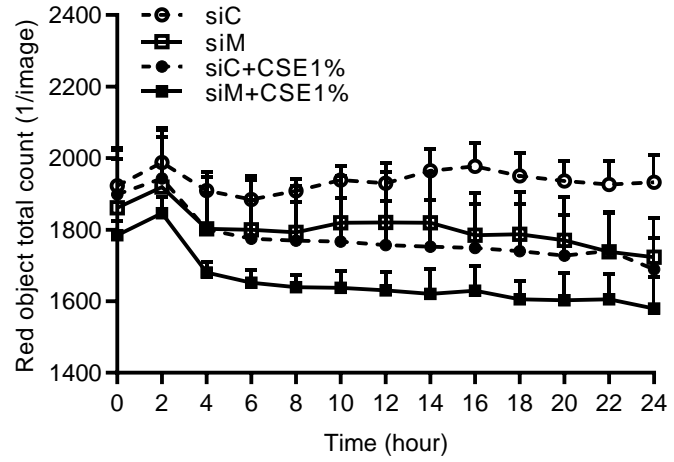

Supplement: Supplementary file 1 [file cells-09-00356-s001.pdf]
